# Perioperative outcomes of zero ischemia radiofrequency ablation-assisted tumor enucleation for renal cell carcinoma: results of 182 patients

**DOI:** 10.1186/s12894-018-0356-1

**Published:** 2018-05-15

**Authors:** Chengwei Zhang, Xiaozhi Zhao, Suhan Guo, Changwei Ji, Wei Wang, Hongqian Guo

**Affiliations:** 1 Department of Urology, Nanjing Drum Tower Hospital, Medical School of Nanjing University, 321 Zhongshan Rd., Nanjing, 210008 People’s Republic of China; 2 School of Public Health, Nanjing Medical University, Nanijng, 210029 People’s Republic of China

**Keywords:** Zero ischemia, Radiofrequency ablation, Tumor enucleation, Renal cell carcinoma, Nephrometry scoring systems

## Abstract

**Background:**

To evaluate the perioperative outcomes of zero ischemia radiofrequency ablation-assisted tumor enucleation.

**Methods:**

Patients undergoing zero ischemia radiofrequency ablation-assisted tumor enucleation were retrospectively identified from July 2008 to March 2013. The tumor was enucleated after RFA treatment. R.E.N.A.L., PADUA and centrality index (C-index) score systems were used to assess each tumor case. We analyzed the correlation of perioperative outcomes with these scores. Postoperative complications were graded with Clavien-Dindo system. Multivariate logistic regression analyses were used to assess risk of complications.

**Results:**

Among 182 patients assessed, median tumor size, estimated blood loss, hospital stay and operative time were 3.2 cm (IQR 2.8–3.4), 80 ml (IQR 50–120), 7 days (IQR 6–8) and 100 min (IQR 90–120), respectively. All three scoring systems were strongly correlated with estimated blood loss, hospital stay and operative time. We found 3 (1.6%) intraoperative and 23 (12.6%, 13 [7.1%] Grade 1 and 10 [5.5%] Grade 2 & 3a) postoperative complications. The median follow-up was 55.5 months (IQR 45–70). Additionally, the complexities of R.E.N.A.L., PADUA and C-index scores were significantly correlated with complication grades (*P* < 0.001; *P* < 0.001; *P* < 0.001; respectively). As the representative, R.E.N.A.L. score was an independent predictive factor for postoperative complications and patients with a high complexity had an over 24-fold higher risk compared to those with a low complexity (OR 24.360, 95% CI 4.412–134.493, *P* < 0.001).

**Conclusions:**

Zero ischemia radiofrequency ablation-assisted tumor enucleation is considered an effective nephron-sparing treatment. Scoring systems could be useful for predicting perioperative outcomes of radiofrequency ablation-assisted tumor enucleation.

## Background

Increasing numbers of small and incidental renal tumors have been detected with the enhancement of imaging technology. The estimated incidence of renal cancers is 5% among all tumors for males and 3% for females [1]. Nephron-sparing surgery (NSS) has been the recommended method to treat cT1a and T1b renal tumors to preserve renal function [2, 3]. However, traditional NSS is considered to have some concerns, including hemostasis, tumor margin status, the collecting system invasion, renal vasculature clamping and hypothermia deployment.

Excision of the tumor with a substantial margin of normal parenchyma is the standard technique in partial nephrectomy and may reduce the risk of local recurrence [4]. To preserve more kidney parenchyma and avoid major bleeding, simple tumor enucleation (TE) was introduced in 2006. TE is a safe and acceptable treatment for NSS [5]. Moreover, the oncologic result with TE is similar to that with radical nephrectomy for treatment of both T1a and T1b renal cell carcinoma (RCC) [6].

As a result of hilar clamping, renal function will be influenced to a certain extent after ischemia with traditional NSS or simple TE, which is more important to the patients who suffer from solitary kidney. Radiofrequency ablation (RFA) has been used in medical field for more than 75 years [7]. The combination of RFA and NSS began in 2003, with no need for clamping the renal pedicle [8]. We reported our technique of RFA-assisted TE for renal tumors in 2012. Hemorrhage can be controlled to some extent and ischemia can be avoided to better protect renal function [9]. Therefore, we can achieve zero ischemia within our TE process.

Nephrometry scoring systems were recently created to predict surgical outcomes after partial nephrectomy. The R.E.N.A.L. and PADUA nephrometry scoring systems contain analogous elements, including tumor size, tumor depth, proximity or aggressiveness to the collecting system, tumor position (anterior or posterior plane) and tumor location in terms of polarity or relation to renal hilum [10, 11]. Differently, The centrality index (C-Index) indicates tumor size and proximity relative to the renal hilum, which provides a measurement of tumor centrality [12]. Both R.E.N.A.L and C-index were found associated with decreased estimated glomerular filtration rate (eGFR) after partial nephrectomy [13, 14]. Satasivam et al.’s report figured out R.E.N.A.L score would predict histological features of tumor aggressiveness [15]. As a vital variable of standard NSS surgery, ischemia time was proved to have strong relationship with all three nephrometry score systems [16, 17]. In spite TE has been widely approved for treatment of RCC, few studies focus on nephrometry scores to evaluate clinical outcomes after TE.

Therefore, we attempt to evaluate the perioperative outcomes of RFA-assisted TE for RCC in our single institute and associate the use of nephrometry scoring systems for predicting the perioperative complications.

## Methods

### Patients

We retrospectively identified consecutive patients who underwent RFA-assisted TE via an open or laparoscopic approach for a single renal tumor in our institution between July 2008 and March 2013. Patients with pathologically confirmed RCC were included. In addition to RFA assisted TE, simple TE and simple RFA are both our choices in the treatment for RCC. In this study all the selected patients were undergoing RFA assisted TE. All patients were informed of the option and all provided signed informed consent to be in the study, which was approved by the local ethics committee.

### Measurements

Preoperatively, all tumors seen on enhanced computerized tomography (CT) or magnetic resonance imaging (MRI) were scored by three senior urologists with different degrees of expertise in terms of the scoring systems. Final scores had interobserver concordance. Tumor stage was determined by the 2010 tumor-node-metastasis classification [4]. Ultrasonography and CT or MRI of the abdomen were performed preoperatively, as was chest X-ray, testing for serum creatinine level and other examinations. The eGFR was calculated by the modified Modification of Diet in Renal Disease equation (MDRD) before and after surgery [18]. Estimated blood loss, operative time and hospital stay were recorded.

Complexity levels of each nephrometry scoring system were defined as follows: R.E.N.A.L Scores ranged from 4 to 12 points. A score of 4, 5 or 6 indicated a lesion of low complexity, and 10, 11 or 12 indicated the highest complexity [10]. PADUA scores ranged from 6 to 14. Tumor with a score of 6 or 7 was considered as low complexity while a score above 9 was high complexity [11]. For the C-index system, tumors were separated into 2 categories of greater than 2.5 (low complexity) or less than 2.5 (high complexity) [13].

### Zero ischemia RFA-assisted TE

Our zero ischemia RFA-assisted TE technique was previously described [9]. All patients were under general anesthesia. The laparoscopic or open approach was via a retroperitoneal or transperitoneal route. The kidney was completely separated from perirenal fat and the renal pedicle was isolated. We localized the tumor by direct vision or intraoperative open or laparoscopic ultrasonography. Before RFA the tumor was biopsied percutaneously (17-gauge TruCore).

The electrode was inserted into the tumor via a percutaneous or laparoscopic approach, under the guidance of intraoperative ultrasonography. RFA was performed by the Cool-tip system, which was controlled by a feedback algorithm. One to three cycles were used, depending on tumor size and depth.

TE was performed with an open or laparoscopic approach with the renal hilum not clamped. Toward the pseudocapsule (PS), we incised the kidney capsule next to the lesion. The surgical plane was determined by the surgeon’s choice. Blunt dissection was used to enucleate the tumors. The rim of the normal renal parenchyma was not visible. Bleeding control involved bipolar coagulation with a 1-cm electrode for several minutes. The parenchymal defect remained open but covered with fibrin glue; the opening of the calyces was ligated by running or single suture with 3-zero monofilament. A single surgeon (HG) performed all surgeries.

### Follow-up

The follow-up protocol at our institution comprised a clinical visit and physical examination, as well as contrast enhanced CT at 7 days, 3, 6 months and then every 6 months thereafter sequentially. Patients with renal insufficiency or contrast agent allergy were followed by enhanced MRI.

### Statistical analysis

Data are presented as mean (SD), median (interquartile range [IQR]) or number (%). All demographic data, including continuous and variables, was analyzed by independent chi-square test. Multivariate logistic regression analysis was used to determine variables predicting perioperative incidences of complications. Odds ratios (ORs) and 95% confidence intervals (95% CIs) were calculated. Spearman’s nonparametric method was used for correlation analysis because of nonnormal distribution of scores. All statistical analyses involved use of SPSS 18.0 (SPSS Inc., Chicago, IL). *P* < 0.05 was considered statistically significant.

## Results

Patients’ demographics are demonstrated in Table 1. We identified 182 patients with perioperative imaging data (125 men [68.7%]; mean [SD] age 57.6 [SD 11.0]; mean body mass index 23.3 kg/m^2^ [SD 4.2]); the approach was laparoscopic for 170 patients (93.4%) and open for 12 (6.6%); 115 (63.2%) patients suffered from American Society of Anesthesiologists (ASA) scores of I or II and 67 (36.8%) was scores of III. The median (IQR) tumor size was 3.2 cm (2.8–3.9) and most (73.1%, *n* = 133) were more than 50% exophytic. The median (IQR) operative time was 100 min (90–120 min), median estimated blood loss 80 ml (50–120 ml) and median hospital stay 7 days (6–8 days). No residual tumor was found on enhanced CT or MRI after surgery. Additionally, for most patients (n = 133, 73.1%), tumors were clear-cell RCC on histopathology. No viable tumor cells were identified on the parenchymal side and the PS was undamaged in all cases. The median follow-up was 55.5 months (IQR 45–70). Totally there were 11 deaths occurring during the follow-up period, in which 2 were related to renal cancer. Distant metastasis developed in five patients at 18 and 35 months after surgery and they died at 38 and 55 months after surgery, respectively. Three patients suffered from lung metastasis and the other two were bone metastasis.

**Table 1:** Characteristics of patients undergoing radiofrequency ablation (RFA)-assisted tumor enucleation (TE) for renal cell carcinoma (*n* = 182)

Age, years, mean (SD)	57.6 (11.0)
Male gender	125 (68.7)
Body mass index, kg/m^2^, mean (SD)	23.3 (4.2)
Operative time, min, median (IQR)	100 (90–120)
Estimated blood loss, ml, median (IQR)	80 (50–120)
Hospital stay, days, median (IQR)	7 (6–8)
Tumor size, cm, median (IQR)	3.2 (2.8–3.9)
Right tumor laterality	102 (55.7)
Follow-up, months, median (IQR)	55.5 (45–70)
ASA score	
< II	115 (63.2)
> III	67 (36.8)
eGFR, ml/min/1.73m^2^, mean (SD)	
Pre-operation	64.3 (18.1)
12 months post-operation	60.8 (17.3)
Tumor location	
> 50% exophytic	133 (73.1)
< 50% exophytic	41 (22.5)
Entirely endophytic	8 (4.4)
Tumor position	
Anterior	85 (46.7)
Posterior	97 (53.3)
Surgical approach	
Laparoscopic	170 (93.4)
Open	12 (6.6)
Histology, no. patients	
Clear cell renal cell carcinoma	133
Papillary	13
Oncocytoma	10
Angiomyolipoma	15
Chromophobe renal cell cancer	8
Unclassified renal cell cancer	3

Data are no. (%) unless indicated. *IQR* Interquartile range, *ASA* American Society of Anesthesiologists, *eGFR* Estimated glomerular filtration rate

After calculating tumor scores on preoperative imaging, we associated the R.E.N.A.L., PADUA and C-Index scores with some clinical variables (Table 2)**.** For all three scoring systems, in which R.E.N.A.L. score complexity played the most significant role (*P* < 0.001), estimated blood loss, operative time and hospital stay but not eGFR change differed by score complexity. All scores and their complexities were strongly correlated with estimated blood loss, operative time and hospital stay (Table 3). However, correlation coefficients with eGFR change in absolute value or percentage were less than 0.2, suggesting a weak correlation.

**Table 2:** Association between nephrometry scores and clinical outcome variables

Nephrometry scores	No. (%)	Estimated blood loss, ml, median (IQR)	Operative time, min, median (IQR)	Hospital stay, days, median (IQR)	Change in eGFR, median (IQR)
R.E.N.A.L. score					
Low (4–6)	132	70 (50,100)	100 (90,110)	6 (5.25,8)	−4.05 (−6.88,-1.3)
Moderate (7–9)	43	120 (75,160)	120 (100,130)	8 (6,9)	−3.7 (−5.3,−0.9)
High (10–12)	7	210 (170,250)	150 (120,160)	10 (8,10)	-0.9 (− 3.3,1.3)
*P* value		**< 0.001**	**< 0.001**	**< 0.001**	0.136
PADUA score					
Low (6–7)	108	70 (50,90)	100 (90,110)	6 (5.25,8)	− 4.2 (− 6.72,-1.3)
Moderate (8–9)	64	100 (61.25,148.75)	102.5 (90,127.5)	8 (6,9)	− 3.4 (− 6.05,-1.12)
High (10–13)	10	190 (127.5220)	130 (103.75,156.25)	8.5 (6.5,10)	−1.25 (− 3.35,1.95)
*P* value		**< 0.001**	**0.002**	**0.005**	0.112
C-index score					
Low (> 2.5)	116	70 (50,100)	100 (90,113.75)	6 (5,8)	− 3.8 (− 6.72,-0.95)
High (< 2.5)	66	102.5 (75,162.5)	102.5 (95,130)	7.5 (6,9)	− 3.75 (− 5.98,-0.9)
*P* value		**< 0.001**	**0.005**	**0.006**	0.921

Boldface means the data is significant (*P* < 0.05)

**Table 3:** Correlation between nephrometry scores and clinical outcomes

Tumor characteristics and scores	Estimated blood loss	Operative time	Hospital stay	Absolute change in eGFR	Percentage change in eGFR
R.E.N.A.L. score	0.438**	0.252**	0.210*	0.072	0.077
PADUA score	0.373**	0.264**	0.241**	0.087	0.105
C-Index Score	0.407**	0.311**	0.203*	0.007	0.035

Data are Spearman correlation coefficients, ρ

*P < 0.05

**P < 0.001

We evaluated perioperative complications, found 3 intraoperative complications and 23 postoperative ones. All the postoperative complications were classified by the Clavien-Dindo grading system, which included 17 (74%) Grade 1–2 complications (Grade 1: 18, Grade 2: 1) and 6 (26%) Grade 3a complications (Table 4). The major complications (Grade 3a) contained urinary leakage and perinephric urinoma. At the same time, we identified strong correlation of complexities between postoperative complications and all the three systems (*P* < 0.001, Table 5), which meant high systems score was significantly associated with high incidence of complications. R.E.N.A.L. score had a most significant correlation coefficient (ρ = 0.376). To evaluate potential preoperative risk factors associated with postoperative complications, multivariate logistic regression analysis was performed. As the representative of score systems, R.E.N.A.L. score was the only independent predictive factor of the occurrence of postoperative complications (Table 6). Patients with a high complexity (R.E.N.A.L. score 10–12) had an over 24-fold higher risk compared with those with a low complexity (R.E.N.A.L. score 4–6).

**Table 4:** Perioperative complications characteristics

Complications	No. patients
Intraoperative complications	3
Blood loss requiring transfusion	2
Conversion	1
Postoperative complications	23
Clavien-Dindo Grade 1	13
Pain	10
Hematuria	2
Renal infarction	1
Clavien-Dindo Grade 2	4
Limited hematoma	1
Postoperative Fever	3
Clavien-Dindo Grade 3a	6
Urinary leakage (stent)	5
Perinephric urinoma (drainage)	1

**Table 5:** Correlation between nephrometry score complexity and postoperative complication grade

Nephrometry scores	Complication Grade			ρ^a^	*P* value
	Absent	Grade 1	Grade 2 and 3a		
R.E.N.A.L. score					
Low (4–6)	125	4	3	0.376	**< 0.001**
Moderate (7–9)	31	7	5		
High (10–12)	3	2	2		
PADUA score					
Low (6–7)	103	3	2	0.372	**< 0.001**
Moderate (8–9)	53	7	4		
High (10–13)	3	3	4		
C-Index score					
Low (> 2.5)	110	2	4	0.290	**< 0.001**
High (< 2.5)	49	11	6		

^a^pearman correlation coefficients

Boldface means the data is significant (*P* < 0.05)

**Table 6:** Multivariate logistic regression predicting incidences of postoperative complications in patients undergoing RFA-assisted TE

Variable	OR	95% CI	*P* value
Age	0.997	0.954–1.042	0.897
Gender	1.875	0.512–6.868	0.343
BMI	1.955	0.672–6.118	0.169
ASA score	1.817	0.735–5.160	0.278
Laterality	2.499	0.657–9.501	0.179
Surgical approach (laparoscopic vs open)	1.744	0.832–2.011	0.461
R.E.N.A.L Score			
Low (4–6)	Reference		
Moderate (7–9)	7.062	2.566–19.436	**< 0.001**
High (10–12)	24.360	4.412–134.493	**< 0.001**

*OR* Odds ratio, *95% CI* 95% confidence interval, *BMI* Body mass index, *ASA* American Society of Anesthesiologists

Boldface means the data is significant (*P* < 0.05)

## Discussion

We succeeded to perform zero ischemia RFA-assisted TE in 182 patients. We evaluated the follow-up of our RFA-assisted TE and the association of these scoring systems with perioperative outcomes. All three scoring systems were strongly correlated with estimated blood loss, hospital stay and operative time. The complexity of the scoring systems was significantly associated with postoperative complication grades. Additionally, R.E.N.A.L. scores were an independent predictive factor for postoperative complications and patients with a high complexity had an over 24-fold higher risk compared to those with a low complexity (OR 24.360, 95% CI 4.412–134.493, *P* < 0.001). Zero ischemia RFA-assisted TE is considered a safe and effective nephron-sparing treatment. Scoring systems could be useful for predicting perioperative outcomes of RFA-assisted TE.

Simple TE has been found a safe and acceptable NSS treatment. Carini et al. evaluated the safety and efficacy of simple TE as a conservative treatment in the early twentieth century: among 232 patients undergoing TE for sporadic, unilateral, pathologically confirmed pT1a RCC, no major complications were found [5]. Likewise, TE was associated with the same progression-free survival and cancer-specific survival as with racial nephrectomy (RN) for both T1a and T1b RCC [6]. In terms of adverse events, the rate was 16%, with only 3% needing re-intervention [19]. TE and RN showed oncologic equivalence in a large cohort (about 1000 patients undergoing RN and 500 TE) in 16 centers [20].

The width of surgical margin seems the most striking difference between TE and traditional NSS. Continuous PS determines the oncologic safety of TE. In general, TE is performed by blunt dissection by using the natural cleavage plane between the tumor and the normal parenchyma. Among 90 consecutive patients undergoing TE, 67% tumors were intact and uninvaded. Although the remaining patients showed signs of penetration within layers, only 6 showed penetration on the perirenal fat tissue side. The surgical margin was negative after TE in all cases [21].

RFA is considered a minimally invasive technique to treat renal tumor. In the past 10 years, percutaneous RFA was an effective treatment for patients, who survived with a small renal mass but had poor surgical condition [3]. Our institute started to treat renal tumors with RFA in 2005 [22]. We have identified that R.E.N.A.L. score is independently associated with occurrence of complications after RFA [23].

RFA-assisted TE has many advantages as compared with simple RFA or NSS. We have achieved zero ischemia during resection of renal tumors with RFA-assisted TE, which maximizes the prevention or delay of decreased renal function. Huang J reported a randomized clinical trial in 2016 and compared the renal functional outcome between RFA assisted TE and conventional laparoscopic partial nephrectomy. Results showed zero ischemia RFA assisted TE presented better renal function preservation. Our results in this article proved that the functional outcomes were also associated with the nephrometry scores [24]. With RFA as a single procedure, our technique allows surgeons to remove the entire tumor, which can provide an accurate pathological result to identify positive or negative surgical margins. According to our previous report, we have proved the oncological safety for the technique of RFA assisted TE. No patient showed positive surgical margins. Microscopy revealed that the pseudocapsule was intact in all cases and no viable tumor cells were identified on the parenchymal side of the tumor [9].

Since our technique is performed without hilar clamping, we did not achieve good intraoperative bleeding control. However, we found little incidence of blood transfusion after surgery because of the superiority of RFA for hemostasis. In contrast, the incidence of urinary leakage seemed high as compared with conventional NSS or TE. We found 5 patients with prolonged urinary leakage, which might occur when the tumor was close to the calyces. We found that the nephrometry scoring systems could predict the incidence of complications. As the result, communication and counseling to patients with high nephrometry scores is important. Rosevear et al. similarly showed an association of the R.E.N.A.L. score and complications for patients undergoing partial nephrectomy [25]. Bruner et al. reported an association between R.E.N.A.L. score and urinary leakage after partial nephrectomy [26], which also could be concluded from our research. On the basis of Minervini’s report about TE, PADUA score was associated with complications, especially Clavien-Dindo grade 3 surgical complications [27].

Our study has some limitations. Because of the short follow-up, we cannot evaluate the 5-years OS or CSS of RFA-assisted TE. Secondly, our study was retrospective. Randomized controlled studies about RFA-assisted TE should be performed to study this technique further.

## Conclusions

Zero ischemia RFA-assisted TE is considered an oncologically safe technique to treat renal cancer, for both protect of renal function and low rate of perioperative complications. Nephrometry scoring systems represent a multifactorial approach to evaluate the renal masses and categorize patients undergoing RFA-assisted TE. From the strong relationship we found, scoring systems may give pertinent information about perioperative outcomes. Zero ischemia RFA-assisted TE is an effective option to treat renal carcinoma.

## Abbreviations

95% CI: 95% confidence interval
C-Index: Centrality index
CT: Computerized tomography
eGFR: Estimated glomerular filtration rate
HR: Hazard ratio
MRI: Magnetic resonance imaging
NSS: Nephron-sparing surgery
OR: Odds ratio
PS: Pseudocapsule
RCC: Renal cell carcinoma
RFA: Radiofrequency ablation
RN: Racial nephrectomy
TE: Tumor enucleation
